# Expression Patterns of Glycosylation Regulators Define Tumor Microenvironment and Immunotherapy in Gastric Cancer

**DOI:** 10.3389/fcell.2022.811075

**Published:** 2022-02-15

**Authors:** Fang Li, Kaibin Huang, Chaohu Pan, Yajie Xiao, Qijun Zheng, Keli Zhong

**Affiliations:** ^1^ Department of Gastrointestinal, Shenzhen People’s Hospital, Second Clinical Medical College of Jinan University, Shenzhen, China; ^2^ YuceBio Technology Co., Ltd, Shenzhen, China; ^3^ Zhuhai Institute of Translational Medicine, Zhuhai People’s Hospital (Zhuhai Hospital Affiliated with Jinan University), Jinan University, Zhuhai, China; ^4^ The Biomedical Translational Research Institute, Faculty of Medical Science, Jinan University, Guangzhou, China; ^5^ Department of Cardiovascular Surgery, Shenzhen People’s Hospital, Second Clinical Medical College of Jinan University, Shenzhen, China

**Keywords:** gastric cancer, scoring system, immunotherapy, prognosis, glycosylation

## Abstract

Glycosylation (Glyc) is prevalently related to gastric cancer (GC) pathophysiology. However, studies on the relationship between glycosylation regulators and tumor microenvironment (TME) and immunotherapy of GC remain scarce. We extracted expression data of 1,956 patients with GC from eight cohorts and systematically characterized the glycosylation patterns of six marker genes into phenotype clusters using the unsupervised clustering method. Next, we constructed a Glyc. score to quantify the glycosylation index of each patient with GC. Finally, we analyzed the relationship between Glyc. score and clinical traits including molecular subtype, TME, and immunotherapy of GC. On the basis of prognostic glycosylation-related differentially expressed genes, we constructed the Glyc. score and divided the samples into the high– and low–Glyc. score groups. The high–Glyc. score group showed a poor prognosis and was validated in multiple cohorts. Functional enrichment analysis revealed that the high–Glyc. score group was enriched in metabolism-related pathways. Furthermore, the high–Glyc. score group was associated with the infiltration of immune cells. Importantly, the established Glyc. score would contribute to predicting the response to anti–PD-1/L1 immunotherapy. In conclusion, the Glyc. score is a potentially useful tool to predict the prognosis of GC. Comprehensive analysis of glycosylation may provide novel insights into the epigenetics of GC and improve treatment strategies.

## Introduction

Gastric cancer (GC) is one of the leading contributors to the global cancer disease burden, which has brought heavy burden to societies and families (Siegel et al., 2020). Despite the many treatments available to tackle GC, it still ranks third in tumor-related mortality (Smyth et al., 2020). Previous studies have shown that the onset of GC is due to cumulative mutations in key signaling pathways (Zhang and Zhang, 2017; Yeoh and Tan, 2021). Although cumulative mutations may be part of the cause of GC, glycosylation in GC-related genes is also associated with GC (Fernandes et al., 2020; Aziz et al., 2021). More than half of the proteins in organisms have glycosylation modification, including transcription factors and metabolism-related enzymes (Ferreira et al., 2021; Sun et al., 2021; Zheng et al., 2021). Glycosylation modification affects protein spatial conformation, activity, transportation, and positioning and is widely involved in intercellular recognition, regulation, and signal transduction (Hombu et al., 2021). Protein glycosylation has been proved to play an important role in the occurrence and progression of various metabolic diseases (Dozio et al., 2021) and malignant tumor diseases (Reily et al., 2019). Because protein glycosylation usually occurs in the early stage of the malignant changes, therefore, further exploring the molecular mechanisms of glycosylation regulation will help to identify new GC markers and, thus, improve the prognosis of patients with GC.

Glycosyltransferase and glycosidase are the key enzymes in glycosylation (Glyc) (Munkley and Elliott, 2016). It can transfer the monosaccharide part of its corresponding donor to sugars, lipids, and proteins, thus to perform glycosylation processing on the latter and realize its biology function. According to different glycosidic bonds, proteins can be glycosylated into N-glycosylation, O-glycosylation, glycophosphatidylinositol, and C-mannosylation (Varki, 2017). Mounting evidence demonstrated that glycosylation regulators involved multiple biological processes at the posttranscriptional level and immunomodulatory abnormality. Studies have found that the overexpression of GnT-V in GC cells increased the *β*1,6GLcNAc branching N-glycosylation modification of e-cadherin, inhibited intercellular adhesion and downstream signal transduction, and promoted the invasion and metastasis of tumors (Ihara et al., 2002). Meanwhile, glycosylation of RIPK3 at T467 was found to be crucial for RIPK3-mediated inflammatory response and necrosis pathway (Li et al., 2019). Despite extensive research on glycosylation, clinical research on the relationship between glycosylation and cancer development is inadequate. The investigation of various transcript isoforms derived from glycosylation patterns may provide new insights into the mechanisms of GC tumorigenesis and development. To better understand the relationship between these glycosylation patterns and the prognosis of GC, we constructed a prognostic scoring system on the basis of the glycosylation.

In this study, we integrated the transcriptome data of 1,956 GC samples to comprehensively evaluate the biological patterns derived from glycosylation regulators. We identified two distinct Glyc patterns and found that they were significantly related to cancer-related signaling pathways. Next, we constructed a Glyc. score to quantify the efficacy of different modification patterns in each patient with GC. We revealed that the distribution of Glyc. score was consistent with multiple cohorts, suggesting that this scoring system based on glycosylation plays an important role in predicting the prognosis and immunotherapy of GC.

## Materials and Methods

### Data Source and Preprocessing

**FIGURE 1:**
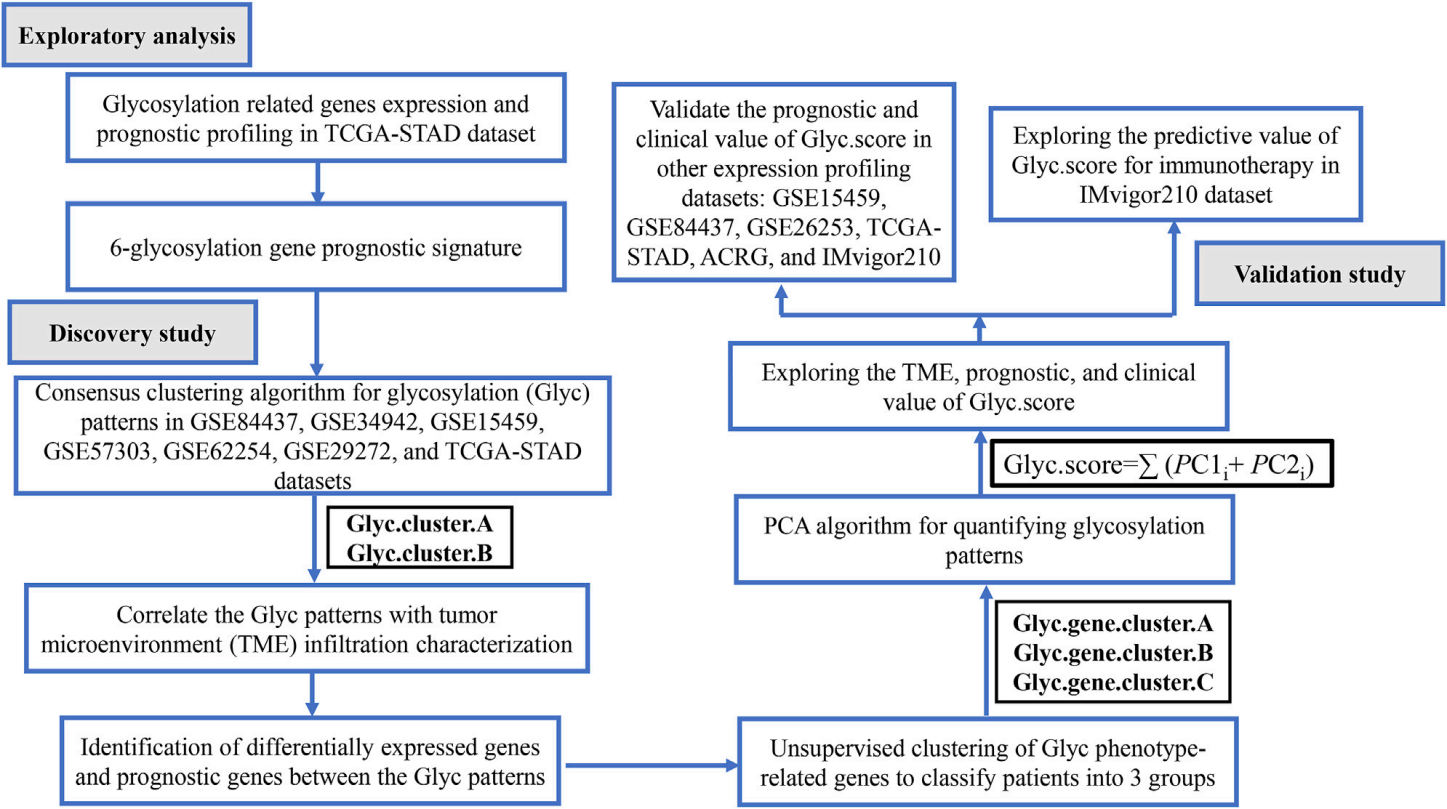
Flow chart of the study.

The workflow of this study is shown in Figure 1. We systematically searched GC-related array datasets from the public databases and selected the GC microarray data since 2010. The GC microarray datasets were recruited from Gene Expression Ominibus (GEO) database (https://www.ncbi.nlm.nih.gov/geo/) with the following criteria: 1) only from Affymetrix platform; 2) GC; 3) the number of patients ≥ 50; 4) with more than 12,000 protein-coding genes. We deleted samples with overall survival (OS) less than 50 days. Finally, GSE84437 (N = 431) (Yoon et al., 2020), GSE34942 (N = 56) (Chia et al., 2015), GSE15459 (N = 191) (Ooi et al., 2009), GSE57303 (N = 70) (Qian et al., 2014), ACRG/GSE62254 (N = 300) (Cristescu et al., 2015), GSE29272 (N = 126) (Wang et al., 2013), and GSE26253 (N = 432) (Lee et al., 2014) microarray datasets were retrieved. One dataset was from The Cancer Genome Atlas (TCGA) (https://portal.gdc.cancer.gov/repository): TCGA-STAD (N = 350). Another immunotherapy dataset was from IMvigor210 (n = 348) (Necchi et al., 2017). We processed the raw data of these datasets using the Robust Multi-array Average method implemented in the affy package for background adjustment, quantile normalization, and final summarization of oligonucleotides per transcript via median polish algorithm (Gautier et al., 2004). The FPKM data were used for differential expression analysis; the FPKM values were transformed into TPM values, which are more similar to the data distribution from microarrays and more comparable between samples analyzed on different platforms (Zhao et al., 2020). Information on the data obtained is summarized in Table 1. Batch effects among different cohorts were removed using the “ComBat” algorithm of the “sva” package in R (Parker et al., 2014). In addition, corresponding clinical information was extracted and manually organized either by directly downloading from the corresponding websites in GEO, IMvigor210, and TCGA or by searching the published primary reports.

**TABLE 1:** Basic information of series used in this study.

Series accession numbers	Platform used	No. of input patients	AJCC_stage	Gender	Mean age, [min, max]	Region	Survivval outcome
GSE84437	Illumina HumanHT-12 V3.0 expression beadchip	431	I: 18; II: 206; III: 209	Female: 137, Male: 296	60.1, [27, 86]	Korea	OS
GSE34942	Affymetrix Human Genome U133 Plus 2.0 Array	56	I: 11; II: 11; III: 19; IV: 13; NA: 2	Female: 20, Male: 36	69, [43, 85]	Singapore	OS
GSE15459	Affymetrix Human Genome U133 Plus 2.0 Array	191	I: 31; II: 29; III: 72; IV: 60	Female: 67, Male: 125	64.4, [23, 92]	Singapore	OS
GSE57303	Affymetrix Human Genome U133 Plus 2.0 Array	70	I: 3; II: 9; III: 41; IV: 17	Female: 18, Male: 52	60.7, [37, 79]	China	OS
ACRG/GSE62254	Affymetrix Human Genome U133 Plus 2.0 Array	300	I: 30; II: 97; III: 96; IV: 77	Female: 101, Male: 199	64, [24, 86]	Asia	OS
GSE29272	Affymetrix Human Genome U133A Array	126	I: 5; II: 5; III: 105; IV: 11	Female: 29, Male: 97	56.7, [23, 73]	United States	OS
GSE26253	Illumina HumanRef-8 WG-DASL v3.0	432	I: 68; II: 167; III: 130; IV: 67	NA	NA	Korea	RFS
TCGA-STAD	Illumina RNAseq	350	I: 52; II: 139; III: 122; IV: 25; NA: 12	Female: 126, Male: 224	67, [35, 90]	NA	OS
IMvigor210	Illumina RNAseq	348	NA	Female: 76, Male: 272	67, [32, 91]	NA	OS

### Selection of Glycosylation Regulators in GC

A gene set of 169 glycosylation regulators was extracted from GlycoGene DataBase (GGDB; https://acgg.asia/ggdb2/) (Supplementary Table S1). Next, use the “Limma” software package in the R statistical software to extract and analyze the TCGA-STAD data and screen out the differentially expressed glycosylation regulators between GC and non-tumor tissues (Ritchie et al., 2015). We set the adjusted *p*-value < 0.05 as a significance threshold. Then, univariate Cox regression analysis was used for differentially expressed glycosylation regulators, and the differentially expressed glycosylation regulators related to the patient’s OS were screened out on the basis of *p* < 0.05.

### Consensus Clustering

Combined analysis on multiple datasets for the six marker genes was conducted to identify glycosylation patterns and classify samples for further analysis, using the unsupervised clustering algorithm. Consensus clustering is a method used for estimating the clustering consensus and assessing stability based on repeated subsampling and clustering, thereby providing the clustering numbers that best fit the data matrix. We used the “ConsensusClusterPlus” package to perform consensus clustering for the gene expression matrix of 1,524 samples and performed 1,000 iterations to ensure the stability of the classification (Wilkerson and Hayes, 2010).

### Differentially Expressed Genes (DEGs) Between the Glycosylation Clusters

After consensus clustering, we classified the samples with different glycosylation patterns into Glyc. cluster.A and Glyc. cluster.B. To identify the phenotype-related DEGs between the two clusters, we used the “limma” package in R, which evaluates the gene expression changes through t-statistics and *p*-values calculated by the empirical Bayes estimation in a linear model. The *p*-values were adjusted using the “Benjamini and Hochberg” method. We set the adjusted *p*-value < 0.05 as a significance threshold.

Construction of a prognostic model by principal component analysis (PCA)

First, we identified 6,352 DEGs with adjusted *p*-value < 0.05 as the significance threshold. Next, to identify which genes are related to the prognosis of GC, we analyzed the DEGs by a univariate Cox regression model. This analysis revealed 2,592 significant prognostic genes (*p* < 0.05). As a result, we performed the consensus clustering again for 2,592 phenotype-related genes from the 1,524 samples to identify the gene clusters among the patients with GC. Finally, all the 2,592 genes were selected as feature genes to construct a PCA scoring system. PCA is one of the most widely used data dimensionality reduction algorithms that preserve the dimensional features with most of the differences (David and Jacobs, 2014). After obtaining the principal component coefficient of each sample, we used a method similar to gene-gene interaction analysis to define the Glyc. score of each sample:

$$\text{Glyc}\text{.Σ}\left(P\text{C}1_{i}+P\text{C}2_{i}\right)$$

where “i” means the significant genes.

### Gene Set Enrichment Analysis (GSEA)

We performed GSEA to identify differences in the enrichment of pathways and biological processes between different IRGs between high–Glyc. score group and low–Glyc. score group. GSEA was conducted using the “clusterProfiler”, “enrichplot”, “patchwork”, and “DOSE” packages in R (Subramanian et al., 2007). We downloaded the gene sets of “c2. cp.kegg.v7.3. symbols” and “h.all.v7.3. symbols” from the MSigDB database for GSEA (http://www.gsea-msigdb.org/gsea/downloads.jsp). A significance level of 0.05 (FDR) was considered to indicate statistical significance.

### Immune Cell Infiltration

We performed the ssGSEA, CIBERSORT, and xCell algorithm methods to calculate the composition of immune cells between the clusters (Bindea et al., 2013; Finotello and Trajanoski, 2018).

### Immunohistochemistry

Tissues of 30 patients with GC and corresponding adjacent tissues were collected to explore the expression of six marker genes in the tissue samples by using immunohistochemical (IHC) staining. IHC staining was performed according to the manufacturer’s instructions.

### Quantitative Reverse Transcription Polymerase Chain Reaction (qRT-PCR) Assays

Total RNA from tissues was isolated using TRIzol (Invitrogen, Canada) reagent, and the specific operation is carried out with reference to the instructions for the operation of the kit. RNA (1 μg) was converted into cDNA using the RevertAid First Strand cDNA Synthesis Kit (Takara, China). qRT-PCR was performed using SYBR Green Mixture (Takara, China) in the ABI StepOne-Plus System (ABI7500, USA). Target gene expression was normalized against GAPDH.

### Statistical Analysis

The normality of the variables was evaluated using the Shapiro–Wilk normality test. We used parametric one-way ANOVA or non-parametric Kruskal–Wallis test to compare between more than two groups. Kaplan–Meier analysis was used to generate survival curves using the “survival” and “survminer” packages, and the cutoff values were determined through the “surv_cutpoint” function in the packages. To calculate the hazard ratios and identify the independent prognostic factors, univariate and multivariate Cox regression analyses were performed using the “survival” package. Subsequently, we employed the area under the ROC curve to measure the prediction accuracy of response to immunotherapy. All statistical analyses were two-sided and considered *p* < 0.05 as the threshold for statistical significance. The statistical results were all analyzed by R (version3.6.2).

## Results

### Identification of Glycosylation Regulators in TCGA-STAD Patients

**FIGURE 2:**
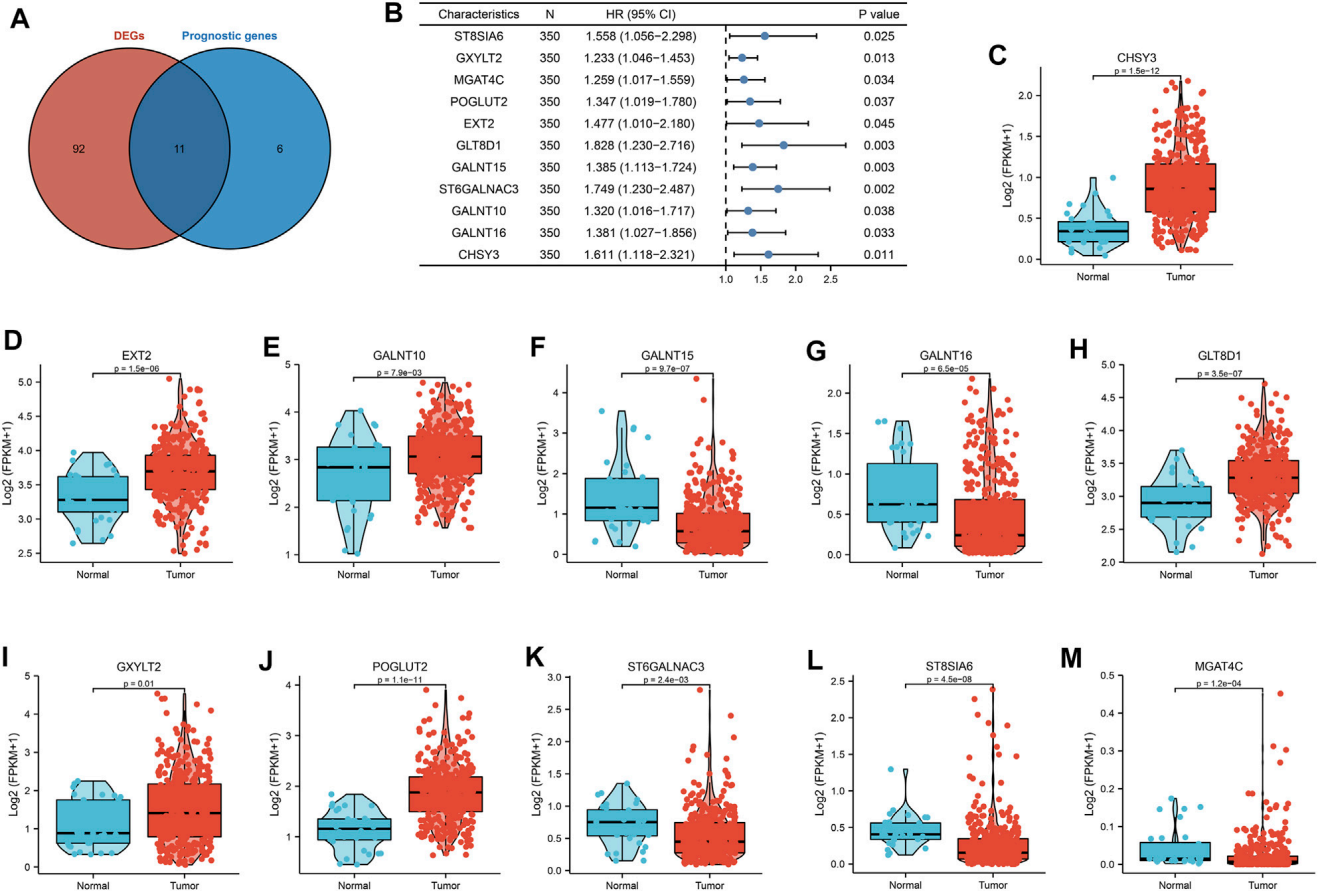
Landscape of expression and prognosis of 11 glycosylation regulators in TCGA-STAD cohort. **(A)** Venn diagram to identify differentially expressed genes between tumor and adjacent normal tissue that were correlated with overall survival. **(B)** Forest plots showing the results of the univariate Cox regression between 11 glycosylation regulators and overall survival in gastric cancer. **(C–M)** The illustration shows the expression distribution of 11 glycosylation regulators between paired normal (blue) and gastric cancer (red) tissues. The boxes indicate the median ±1 quartile, with the whiskers extending from the hinge to the smallest or largest value within 1.5× IQR from the box boundaries.

A total of 169 glycosylation regulators based on the GGDB were identified in this study (Supplementary Table S1). To analyze the expression pattern of glycosylation regulators between the GC and normal control, 103 differentially expressed glycosylation regulators between tumor and adjacent normal tissue in TCGA-STAD dataset were identified (Supplementary Table S2). Next, we sought to evaluate the predictive value of glycosylation regulators for prognosis in GC. Kaplan–Meier log-rank analysis revealed that 17 glycosylation regulators were significantly correlated with OS in patients with GC (Supplementary Table S3). Finally, a total of 11 prognostic Glyc-related DEGs were preserved (Figure 2A). Of these, high expressions of 11 glycosylation regulators were significantly correlated with poor OS in TCGA-STAD dataset (Figure 2B). Besides, the results demonstrated that six glycosylation regulators (GALNT10, EXT2, GLT8D1, GXYLT2, POGLUT2, and CHSY3) were highly expressed in GC than in normal tissues. However, five glycosylation regulators (ST8SIA6, MGAT4C, GALNT15, ST6GALNAC3, and GALNT16) were more lowly expressed in GC than in normal tissues (Figures 2C–M). Ultimately, six glycosylation regulators were included in the following analysis.

### Consensus Clustering Based on the Six Glycosylation Regulators and Functional Enrichment Analysis

**FIGURE 3:**
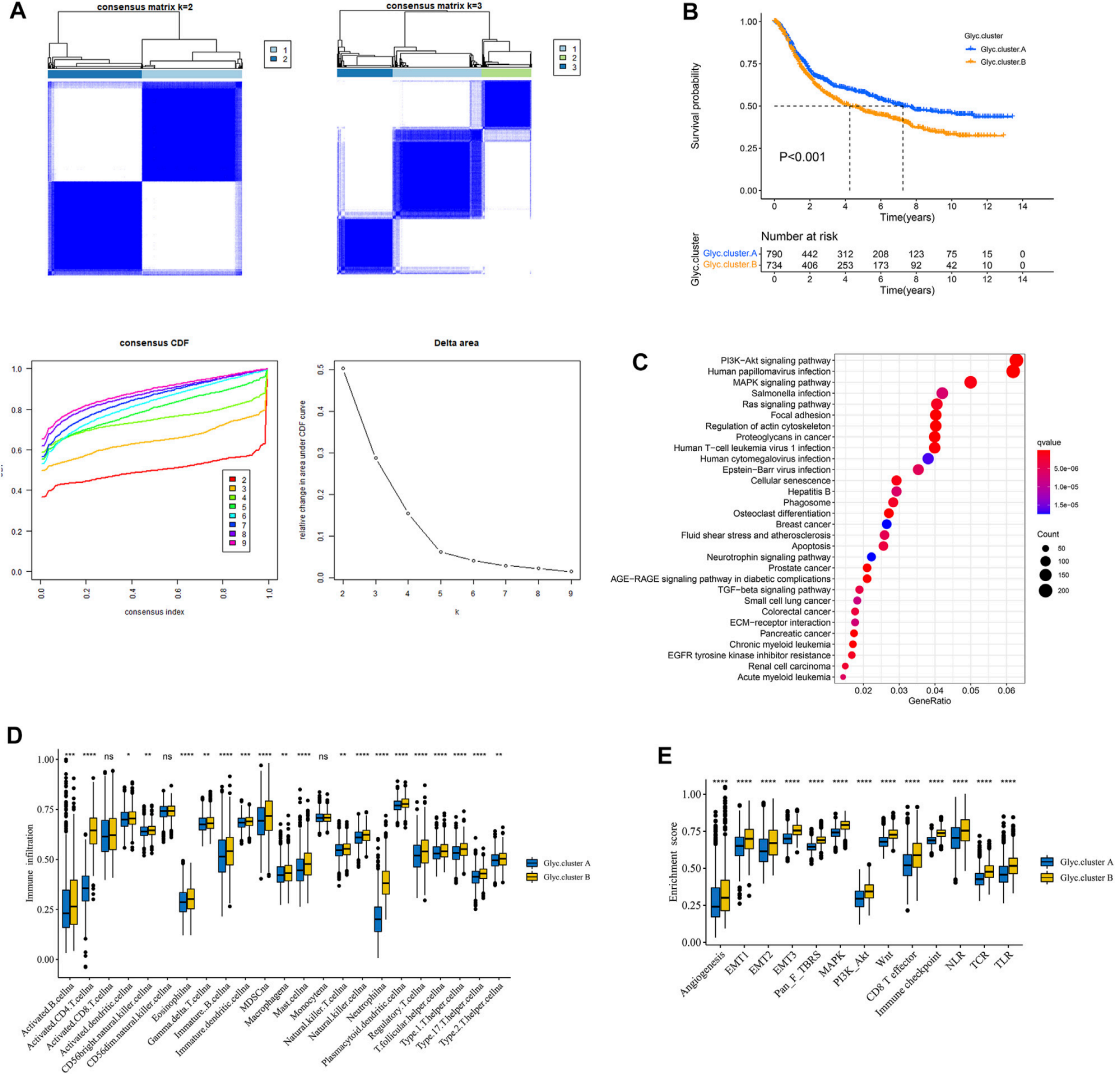
Patterns of glycosylation and functional enrichment analysis. **(A)** Unsupervised consensus clustering for 1,524 gastric cancer patients in a meta cohort (GSE84437, GSE34942, GSE15459, GSE57303, GSE62254, GSE29272, and TCGA-STAD). **(B)** Kaplan–Meier curve showed a significant difference between the two glycosylation (Glyc) clusters. The yellow represented Glyc. cluster **(B)**, and the blue represented Glyc. cluster.A. **(C)** KEGG enrichment analysis. The illustration was used to visualize these biological processes, where red represented activated pathways and blue represented inhibited pathways. **(D)** The abundance of each TME infiltrating cell in two Glyc clusters. The upper and lower ends of the boxes represented the interquartile range of values. The lines in the boxes represented median value, and black dots showed outliers. **(E)** Differences in stroma-activated pathways including EMT, TGF beta, and angiogenesis pathways among two Glyc clusters. The asterisks represented the statistical *p*-value (*p < 0.05; **p < 0.01; ***p < 0.001; ****p < 0.0001).

To classify the glycosylation patterns, we used consensus clustering on the basis of the expression data of the six recognized marker genes. Eventually, we identified two distinct groups termed phenotype Glyc. cluster.A (*n* = 790), and Glyc. cluster.B (*n* = 734) (Figure 3A). Kaplan–Meier analysis showed that the prognosis of Glyc. cluster.B was poor than that of Glyc. cluster.A (Figure 3B, *p* < 0.001). Then, we performed KEGG to compare pathway enrichment between Glyc. cluster.A and Glyc. cluster.B. Hallmark gene sets comprehensively summarized specific well-defined biological processes and displayed high consistency in most published studies. As shown in Figure 3C, compared with Glyc. cluster.A, Glyc. cluster.B was significantly enriched in some immunity- and cancer-related pathways.

Next, we explored whether there is a correlation between the two clusters and immune infiltration levels in GC. Therefore, we used the ssGSEA method to evaluate the composition of immune cells between the two clusters. As shown in Figure 3D, we found that immune cells in the Glyc. cluster.B group exhibited higher expression levels in patients with GC. Subsequent analyses showed that stroma activity was significantly enhanced in Glyc. cluster.B such as the activation of epithelial–mesenchymal transition and angiogenesis pathways, which confirmed our speculation (Figure 3E). Next, we used the CIBERSORT and xCell algorithm methods to calculate the composition of immune cells between the two clusters. As shown in Supplementary Figures 1A,B, we found that immune cells in the Glyc. cluster.B group exhibited higher expression levels in patients with GC.

### Construction of Glyc.gene Signature

**FIGURE 4:**
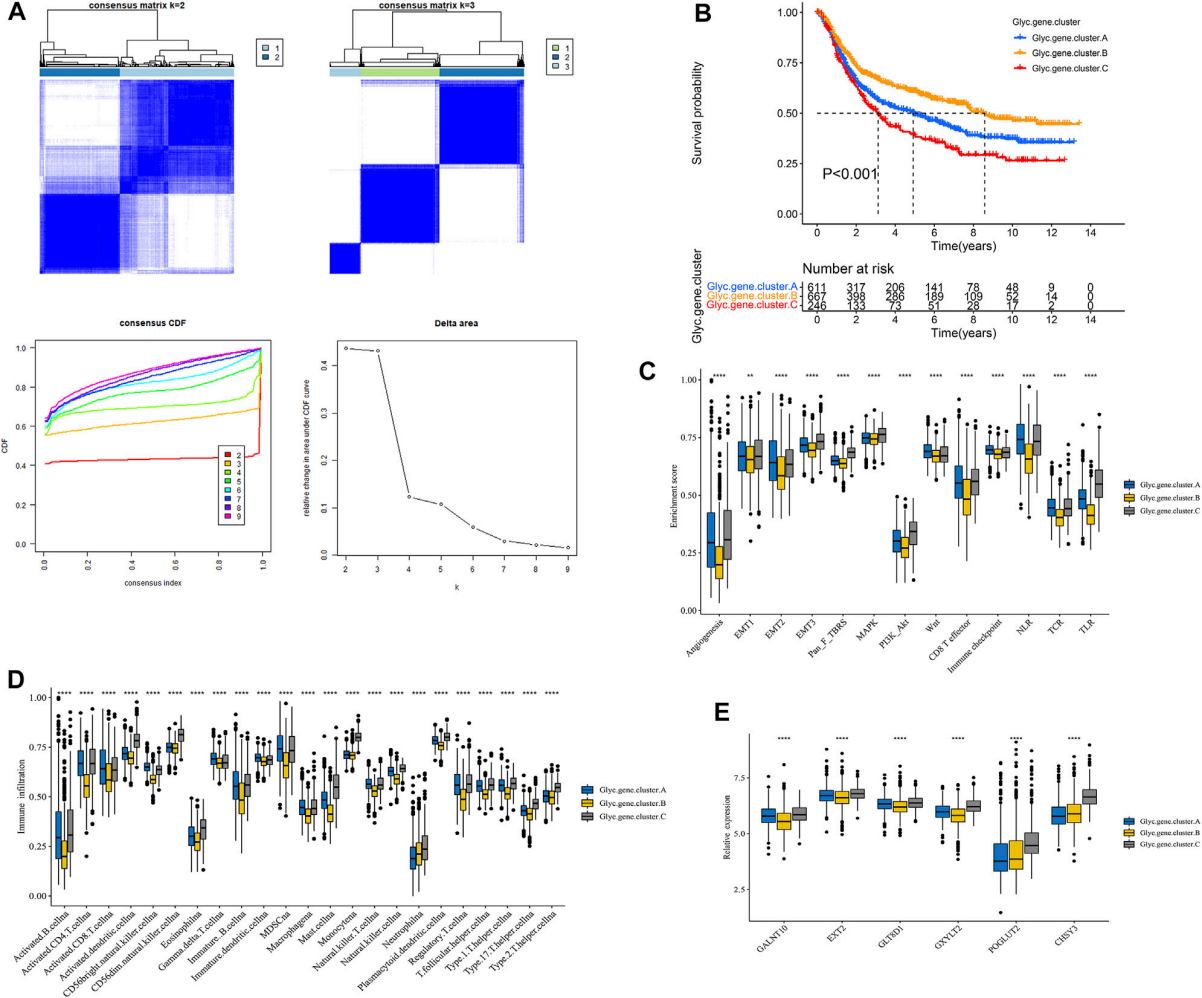
Construction of Glyc. gene signature. **(A)** Unsupervised consensus clustering based on prognostic Glyc-related differentially expressed genes to classify patients into three groups termed Glyc. gene.clusters A, Glyc. gene.clusters B, and Glyc. gene.clusters C. **(B)** Kaplan–Meier curve showed a significant difference between the three Glyc. gene.clusters. **(C)** Differences in stroma-activated pathways including EMT, TGF beta, and angiogenesis pathways among three Glyc. gene.clusters. **(D)** The abundance of each TME infiltrating cell in three Glyc. gene.clusters. The upper and lower ends of the boxes represented the interquartile range of values. The lines in the boxes represented median value, and black dots showed outliers. **(E)** The expression of six Glyc regulators in three Glyc. gene.clusters. The upper and lower ends of the boxes represented interquartile range of values. The lines in the boxes represented median value, and black dots showed outliers. The asterisks represented the statistical *p*-value (*p < 0.05; **p < 0.01; ***p < 0.001; ****p < 0.0001).

First, Glyc. cluster.A group and Glyc. cluster.B group denoted a markedly discrimination each other, suggesting that there are different genes between the two groups (Supplementary Figure S2). We identified 6,352 DEGs with adjusted *p*-value < 0.05 as the significance threshold (Supplementary Table S4). To identify the prognostic genes in GC, we selected these DEGs to perform univariate Cox regression analysis. This analysis revealed 2,592 significant genes (*p* < 0.05, Supplementary Table S5). We further identified the genomic subtypes on the basis of the prognostic genes using the consensus clustering method. There were three subtypes, termed Glyc. gene.cluster.A, Glyc. gene.cluster.B, and Glyc. gene.cluster.C with 661, 667, and 246 samples, respectively (Figure 4A). Kaplan–Meier analysis showed that the prognosis of Glyc. gene.cluster.C was poor than that of Glyc. gene.cluster.A and Glyc. gene.cluster.B (Figure 4B, *p* < 0.001). Next, we explored whether there is a correlation between the three clusters and immune infiltration levels in GC. Therefore, we used the ssGSEA method to evaluate the composition of immune cells between the three clusters. As shown in Figure 4D, we found that immune cells in the Glyc. gene.cluster.C group exhibited higher expression levels in patients with GC. Subsequent analyses showed that stroma activity was significantly enhanced in Glyc. gene.cluster.C such as the activation of epithelial–mesenchymal transition and angiogenesis pathways (Figure 4C). We also used the CIBERSORT and xCell algorithm methods to calculate the composition of immune cells between the three clusters. As shown in Supplementary Figures 3A,B, we found that immune cells in the Glyc. gene.cluster.C group exhibited higher expression levels in patients with GC. Then, we also observed that there were significant differences between the Glyc. gene.cluster.A, Glyc. gene.cluster.B, and Glyc. gene.cluster.C for the N, stage, gender and age, and fustat (Supplementary Figure S4). Moreover, we further analyzed the relationship between three Glyc. gene.clusters and the expression of six glycosylation genes. The result showed the expression of six glycosylation genes in gene Glyc. gene.cluster.C was higher than that in the other groups (Figure 4E). On the basis of the above analyses, most of the samples in Glyc. gene.cluster.A and Glyc. gene.cluster.B could be classified into phenotype Glyc. cluster.A. Samples in Glyc. gene.cluster.C were closely related to phenotype Glyc. cluster.B.

### Construction of Glyc.score

**FIGURE 5:**
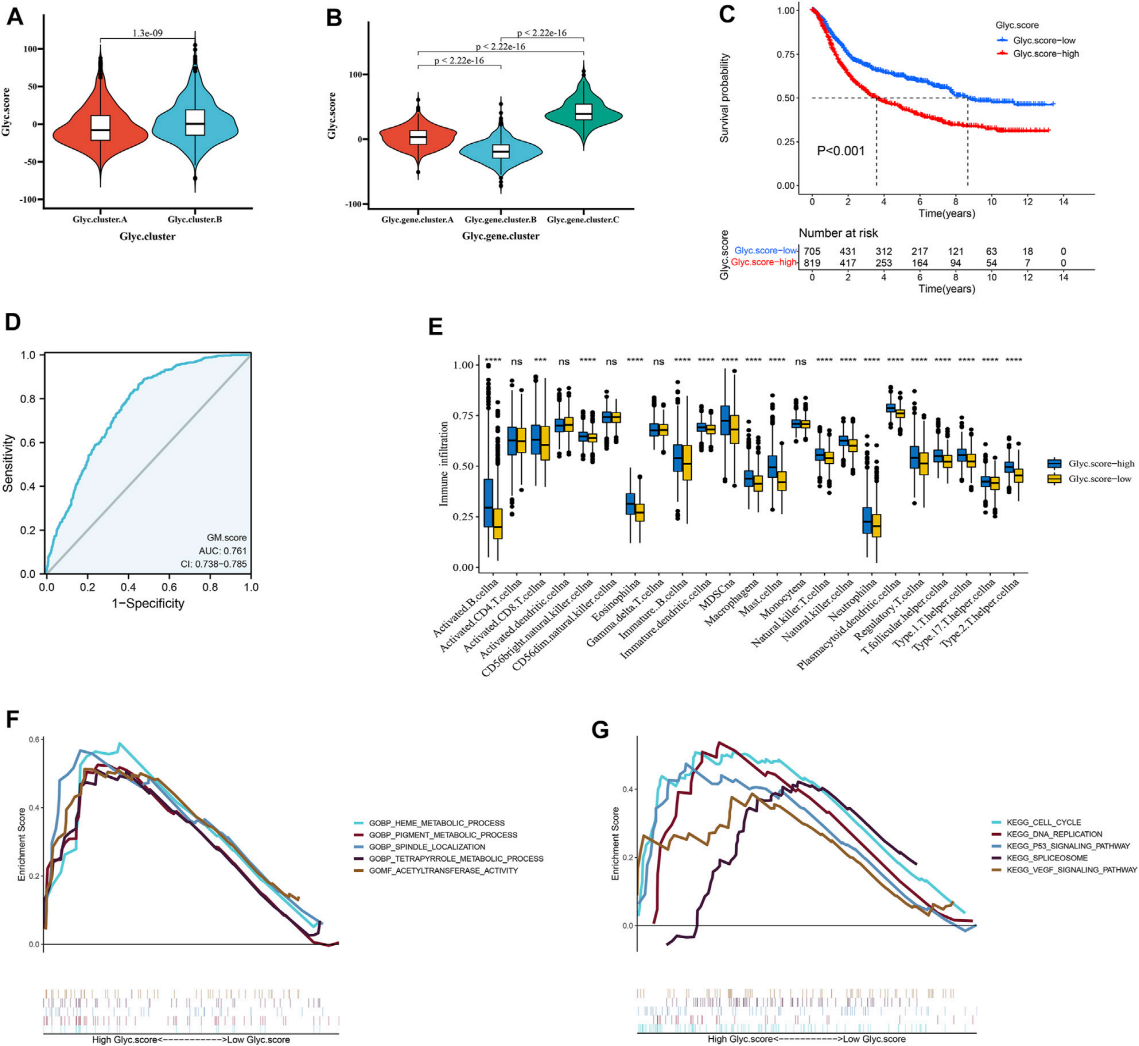
Construction of Glyc. score. **(A)** Differences in Glyc. score among two Glyc. clusters in meta cohort. **(B)** Differences in Glyc. score among three Glyc. gene.clusters in meta cohort. **(C)** Kaplan–Meier curves for high– and low–Glyc. score patient groups. **(D)** The predictive value of Glyc. score in meta cohort. **(E)** The abundance of each TME infiltrating cell in high– and low–Glyc. score groups. The upper and lower ends of the boxes represented the interquartile range of values. The lines in the boxes represented median value, and black dots showed outliers. **(F)** GSEA GO identified high– and low–Glyc. score groups related signaling pathways in gastric cancer. **(G)** GSEA KEGG identified high and low Glyc. score–related signaling pathways in gastric cancer.

We further constructed a scoring system using the PCA method to quantify the Glyc patterns of individual patients with GC. This scoring system was named the Glyc. score. First, we found that the Glyc. score of Glyc. cluster.B was significantly higher than that of Glyc. cluster.A (Figure 5A, *p* < 0.001), and the Glyc. score of Glyc. gene.cluster.C was significantly higher than that of Glyc. gene.cluster.A and Glyc. gene.cluster.B (Figure 5B, *p* < 0.001). Kaplan–Meier analysis revealed that the prognosis of patients with a high Glyc. score was significantly poor than that of patients with a low Glyc. score (Figure 5C, *p* < 0.001). The predictive value of Glyc. score in GC cohorts was 0.761, suggesting that Glyc. score can better predict the prognosis of GC (Figure 5D). We further analyzed the relationship between Glyc. score and immune cells. The result showed immune cells in the high–Glyc. score group exhibited higher expression levels in patients with GC (Figure 5E). Functional enrichment analysis revealed that the high–Glyc. score group was enriched in metabolic- and cancer-related pathways, including cell cycle, heme metabolic process, pigment metabolic process, and tetrapyrrole metabolic process (Figures 5F,G).

### Independent Prognostic Analysis of Glyc.score

**FIGURE 6:**
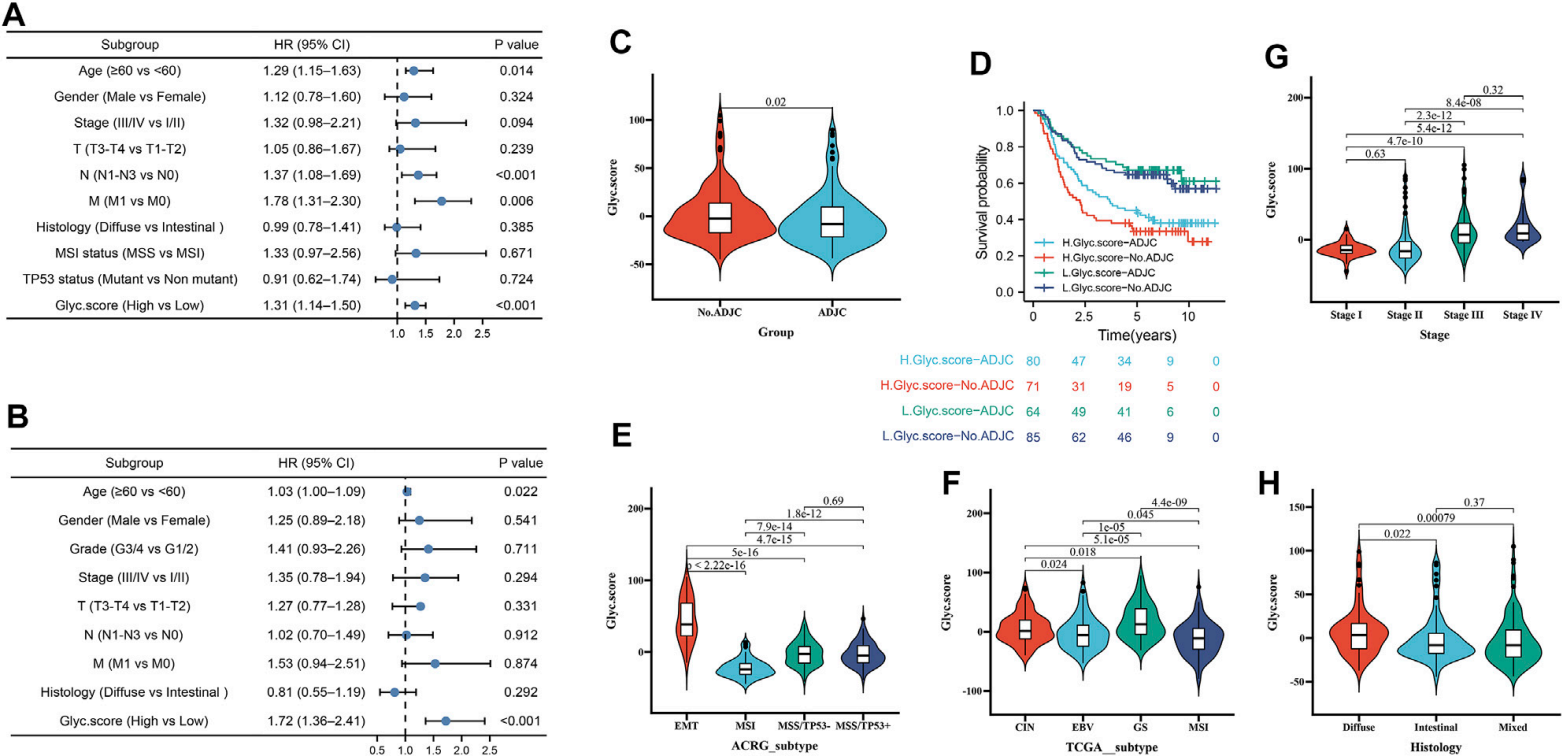
Independent prognostic analysis of Glyc. score. **(A)** Multivariate Cox regression analysis for Glyc. score in ACRG cohort shown by the forest plot. **(B)** Multivariate Cox regression analysis for Glyc. score in TCGA cohort shown by the forest plot. **(C)** Difference in Glyc. score among ADJC and No. ADJC in ACRG cohort. ADJC, adjuvant chemotherapy. **(D)** Kaplan–Meier curves for patients in the ACRG cohort stratified by both receipt of ADJC and Glyc. score. **(E)** Differences in Glyc. score between different ACRG molecular subtypes. The upper and lower ends of the boxes represented interquartile range of values. **(F)** Differences in Glyc. score between different TCGA-STAD molecular subtypes. The upper and lower ends of the boxes represented interquartile range of values. **(G)** Differences in Glyc. score between different stage. **(H)** Differences in Glyc. score between different histology subtypes.

Next, to investigate whether Glyc. score could serve as an independent prognostic factor for GC, we performed multivariate Cox regression analyses. The results indicated that Glyc. score is a robust independent prognostic factor in TCGA cohort (1.72 [1.36–2.41], *p* < 0.001) and ACRG cohort (1.31 [1.14–1.50], *p* < 0.001) (Figures 6A,B). We further analyzed the Glyc. score in No. adjuvant chemotherapy group (ADJC) and adjuvant chemotherapy group, and patients received ADJC had a lower Glyc. score than that patients received No. ADJC (Figure 6C). In addition, both in patients receiving ADJC or not, high Glyc. score had an obvious poor survival than that of patients with a low Glyc. score (Figure 6D). To analyze the clinical significance of Glyc. score, we observed the correlation between Glyc. score and ACRG molecular subtype and TCGA molecular subtype. Consistent with the OS analysis of the ACRG cohort, the Glyc. score of patients with the EMT subtype was relatively high, followed by MSS/TP53+, MSS/TP53−, and finally the MSI subtype (Figure 6E). Meanwhile, consistent with the OS analysis of the TCGA cohort, the Glyc. score of patients with the GS subtype was relatively high, followed by CIN, EBV, and, finally, the MSI subtype (Figure 6F). In addition, we revealed that diffuse histological subtype and advanced patients were significantly associated with a higher Glyc. score, which means that these patients had a poorer clinical outcome (Figures 6G,H).

### Validation of the Prognosis of Glyc. Score in Multiple Cohorts

**FIGURE 7:**
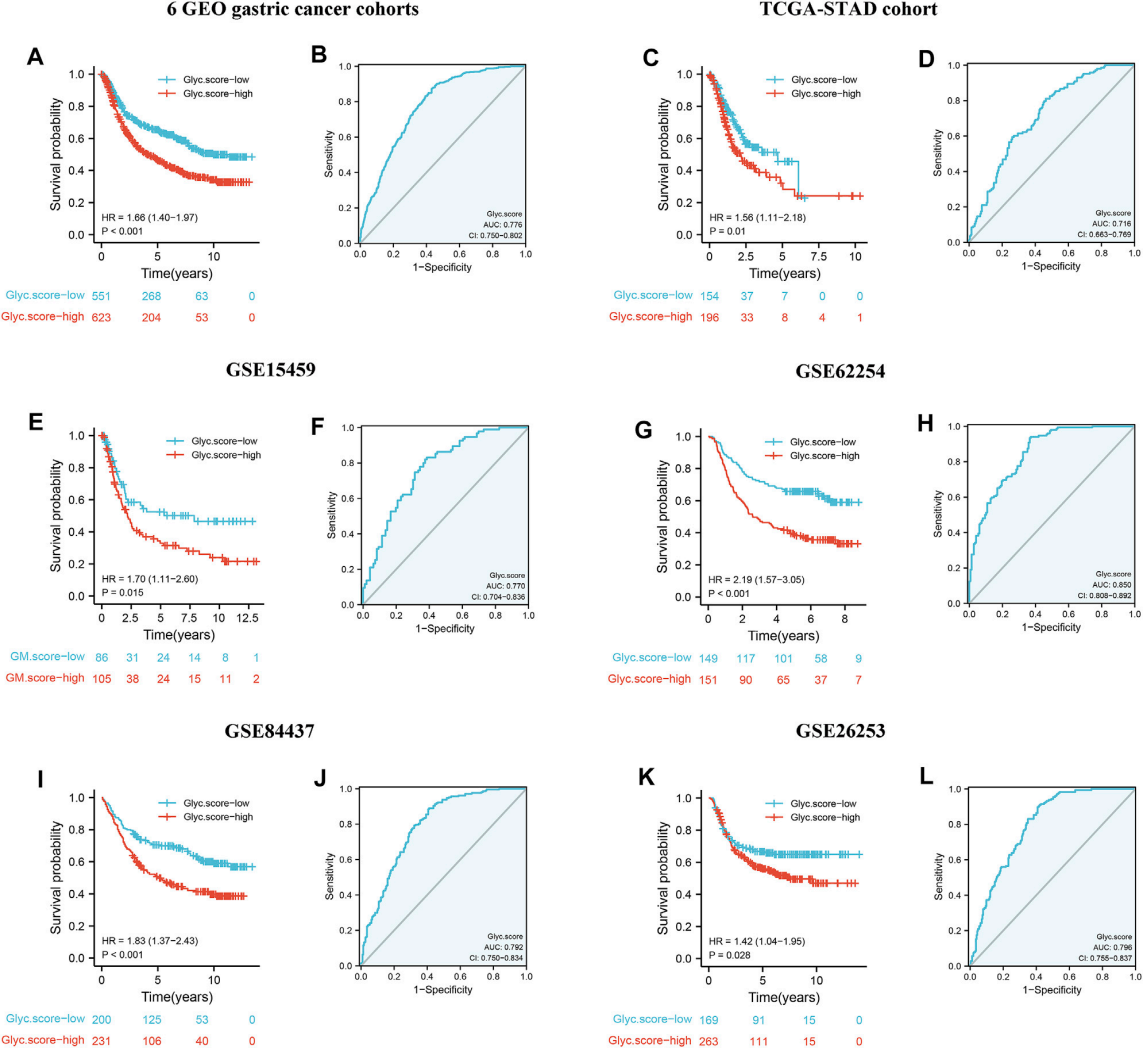
External validation of Glyc. score model. **(A)** Kaplan–Meier curves for high– and low–Glyc. score patient groups in six GEO datasets (GSE84437, GSE34942, GSE15459, GSE57303, GSE62254, and GSE29272). **(B)** The predictive value of Glyc. score in six GEO dataset. **(C)** Kaplan–Meier curves for high– and low–Glyc. score patient groups in TCGA-STAD. **(D)** The predictive value of Glyc. score in TCGA-STAD. **(E)** Kaplan–Meier curves for high– and low–Glyc. score patient groups in GSE15459. **(F)** The predictive value of Glyc. score in GSE15459. **(G)** Kaplan–Meier curves for high– and low–Glyc. score patient groups in GSE62254. **(H)** The predictive value of Glyc. score in GSE62254. **(I)** Kaplan–Meier curves for high– and low–Glyc. score patient groups in GSE84437. **(J)** The predictive value of Glyc. score in GSE84437. **(K)** Relapse-free survival analysis of Glyc. score in GSE26253 cohort. **(L)** The predictive value of Glyc. score in GSE26253.

To further validate Glyc. score in GC, GSE15459, GSE84437, GSE26253, TCGA-STAD, and GSE62254 cohorts were used to measure the Glyc. score. Kaplan–Meier analysis revealed that the prognosis of patients with a high Glyc. score was significantly poor than that of patients with a low Glyc. score (Figure 7A, 1.66 [1.40–1.97], *p* < 0.001). The predictive value of Glyc. score in all GC cohorts except GSE26253 was 0.776 (Figure 7B). Simultaneously, Kaplan–Meier analysis revealed that the prognosis of patients with a high Glyc. score was significantly poor than that of patients with a low Glyc. score in TCGA-STAD (Figure 7C, 1.56 [1.11–2.18], *p* = 0.010), GSE15459 (Figure 7E, 1.70 [1.11–2.80], *p* = 0.015), GSE62254 (Figure 7G, 2.19 [1.57–3.05], *p* < 0.001), and GSE84437 (Figure 7I, 1.83 [1.37–2.43], *p* < 0.001). The predictive value of Glyc. score in TCGA-STAD, GSE15459, GSE62254, and GSE84437 was 0.716 (Figure 7D), 0.770 (Figure 7F), 0.850 (Figure 7H), and 0.792 (Figure 7J), respectively. Finally, Kaplan–Meier analysis revealed that the prognosis of patients with a high Glyc. score was significantly poor relapse-free survival than that of patients with a low Glyc. score in the GSE26253 cohort (Figure 7K, 1.42 [1.04–1.95], *p* = 0.028). The predictive value of Glyc. score in GSE26253 was 0.796 (Figure 7L).

### Glyc. Score Involved in Immunotherapy

**FIGURE 8:**
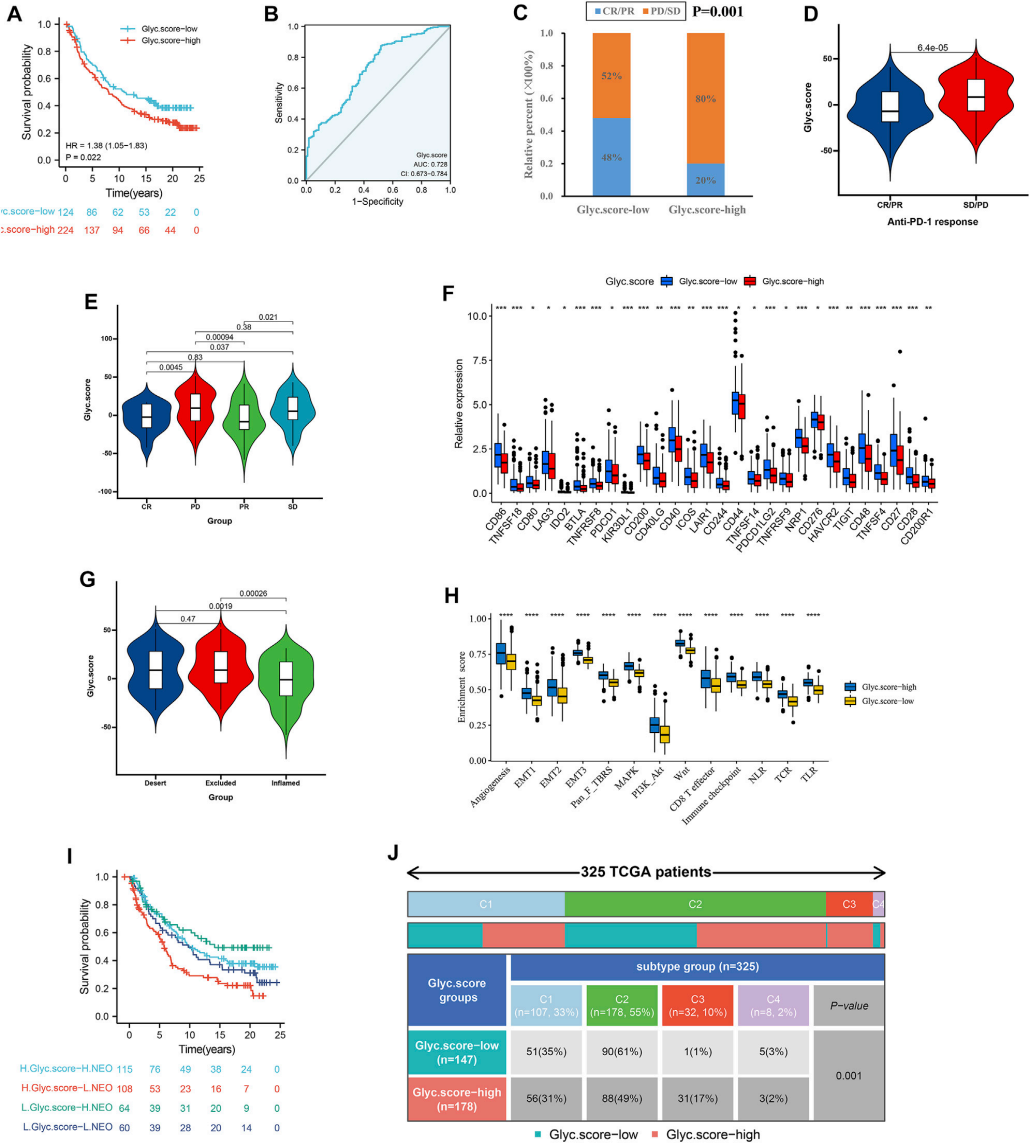
Glyc. score in the role of anti–PD-1/L1 immunotherapy. **(A)** Survival analyses for low– and high–Glyc. score patient groups in the anti–PD-L1 immunotherapy cohort using Kaplan–Meier curves (IMvigor210 cohort). **(B)** The predictive value of Glyc. score in IMvigor210 cohort. **(C)** The proportion of patients with response to PD-L1 blockade immunotherapy in low– or high–Glyc. score groups. SD, stable disease; PD, progressive disease; CR, complete response; PR, partial response. **(D)** Differences in Glyc. score among distinct anti–PD-1 clinical response groups. **(E)** Distribution of Glyc. score in distinct anti–PD-L1 clinical response groups. **(F)** Differences in checkpoint expression between low– and high–Glyc. score groups. **(G)** Differences in Glyc. score among distinct tumor immune phenotypes in IMvigor210 cohort. **(H)** Differences in stromaactivated pathways and abundance of regulatory T cells (considered as immune suppression) between low– and high–Glyc. score groups in anti–PD-L1 immunotherpy cohort. **(I)** Survival analyses for patients receiving anti–PD-L1 immunotherapy stratified by both Glyc. score and neoantigen burden using Kaplan–Meier curves. NEO, neoantigen burden. **(J)** Differences in Glyc. score between immune subtypes (C1, wound healing; C2, IFN-gamma dominant; C3, inflammatory; C4, lymphocyte depleted).

Considering that Glyc. score appears to be associated with tumor microenvironment (TME), we examined the power of the Glyc. score to predict the response of patients to immunotherapy. We found that patients with low Glyc. score exhibited significant clinical benefits and had a markedly prolonged OS in IMvigor210 cohort (Figure 8A). The predictive value of Glyc. score in IMvigor210 cohort was 0.728 (Figure 8B). The 348 patients of the IMvigor210 cohort exhibited different degrees of response to anti–PD-L1 blocker (Table 2). The low–Glyc. score group also showed significantly better therapeutic outcomes (Figure 8C). The CR/PR patients showed the lower Glyc. score than patients with other types of responses (Figures 8D,E). By detecting the expression level of checkpoint, we found that the checkpoint expression in the high–Glyc. score group was lower than in the low–Glyc. score group (Figure 8F). To analyze the immune phenotypes significance of Glyc. score, we observed the correlation between Glyc. score and immune phenotypes. The Glyc. score of patients with the exclusion and desert immune phenotypes was relatively high (Figure 8G). Subsequent analyses showed that stroma activity was significantly enhanced in high Glyc. score such as the activation of epithelial–mesenchymal transition and angiogenesis pathway (Figure 8H). Importantly, we found patients with combination of high Glyc. score and low neoantigen burden showed a poor survival (Figure 8I). We found patients with high Glyc. score were characterized inflammatory phenotype (Figure 8J). In summary, our work strongly indicated that the established Glyc. score would contribute to predicting the response to anti–PD-1/L1 immunotherapy.

**TABLE 2:** Summary of detailed clinical information of IMvigor210 (mUC) cohort.

IMvigor210 cohort	Low (*n* = 124)	High (*n* = 224)	Total (*n* = 348)
Vital status			
Alive	42 (33.9%)	74 (33.0%)	116 (33.3%)
Dead	82 (66.1%)	150 (67.0%)	232 (66.7%)
Gender			
Female	20 (16.1%)	56 (25.0%)	76 (21.8%)
Male	104 (83.9%)	168 (75.0%)	272 (78.2%)
Overall response			
CR	5 (18.5%)	20 (7.4%)	25 (8.4%)
PR	8 (29.6%)	35 (12.9%)	43 (14.4%)
SD	3 (11.1%)	60 (22.1%)	63 (21.1%)
PD	11 (40.7%)	156 (57.6%)	167 (56.0%)
Binary response			
CR/PR	13 (48.1%)	55 (20.3%)	68 (22.8%)
SD/PD	14 (51.9%)	216 (79.7%)	230 (77.2%)
Enrollment IC			
IC0	30 (24.2%)	69 (30.8%)	99 (28.4%)
IC1	51 (41.1%)	81 (36.2%)	132 (37.9%)
IC2	43 (34.7%)	74 (33.0%)	117 (33.6%)
IC level			
IC0	32 (26.0%)	65 (29.0%)	97 (28.0%)
IC1	45 (36.6%)	87 (38.8%)	132 (38.0%)
IC2+	46 (37.4%)	72 (32.1%)	118 (34.0%)
TC Level			
TC0	91 (74.0%)	184 (82.1%)	275 (79.3%)
TC1	11 (8.9%)	11 (4.9%)	22 (6.3%)
TC2+	21 (17.1%)	29 (12.9%)	50 (14.4%)
Immune phenotype			
Desert	22 (19.5%)	54 (31.6%)	76 (26.8%)
Excluded	51 (45.1%)	83 (48.5%)	134 (47.2%)
Inflamed	40 (39.4%)	34 (19.9%)	74 (26.1%)
TCGA cluster			
I	42 (33.9%)	76 (33.9%)	118 (33.9%)
II	46 (37.1%)	49 (21.9%)	95 (27.3%)
III	16 (12.9%)	53 (23.7%)	69 (19.8%)
IV	20 (16.1%)	46 (20.1%)	66 (19.0%)

### Validation the Expression of Six Marker Genes by qRT-PCR and IHC

**FIGURE 9:**
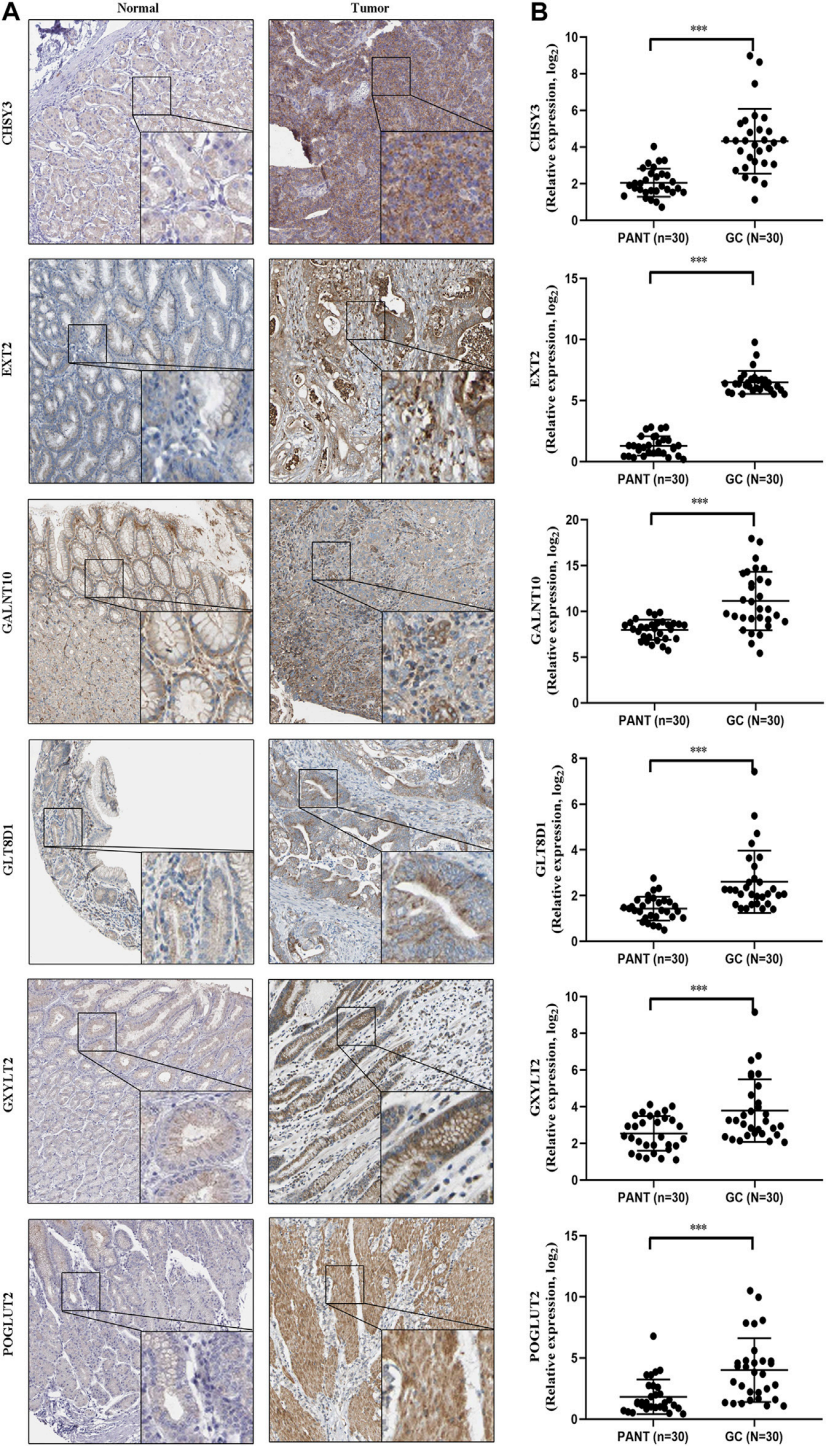
The expression of six marker genes in GC. **(A)** Representative immunohistochemistry images of six marker genes expression in normal tissues and GC tissues. **(B)** Six marker genes mRNA levels are shown for the GC and normal tissues.

Next, to further validate six marker genes in GC, we measured the six marker genes protein level by immunohistochemistry, and the result showed that, compared with the normal group, the six marker genes protein level was significantly higher in the GC group (Figure 9A). In addition, RT-qPCR was used to detect the six marker genes mRNA expression in GC. Compared with the normal group, the six marker genes mRNA level was significantly higher in the GC group (Figure 9B).

## Discussion

Glycosylation implicated in the occurrence and development of cancer. The immune system mainly recognizes the forms of tissue damage and the presence of pathogens. Damage-related molecular patterns are often based on foreign polysaccharides and glycoconjugates, which induce the release of pro-inflammatory cytokines and the activation of immune cells (Khan et al., 2018). However, proinflammatory signaling is inhibited by the autocorrelation molecular pattern of host cells through the expression of specific polysaccharide antigens, and glycosylation modification of proteins may be involved in the regulation (Cadena et al., 2019). In addition to its direct effect on immunosuppression, glycosylation can also indirectly promote immune evasion by enhancing immune checkpoint (Seeling et al., 2017). In this study, we mainly focused on the glycosylation regulators and classified them into two Glyc-related clusters on the basis of the expression profile of six marker genes. Next, we identified three genomic subtypes on the basis of 2,592 prognostic Glyc-related DEGs and constructed the Glyc. score to quantify the Glyc and predict the prognosis and immunotherapy of GC.

From a global perspective, the whole process of clustering and the Glyc. score showed consistency and accuracy. The samples in the high–Glyc. score group were mostly associated with phenotype Glyc. cluster.B and Glyc. gene.cluster.C. The higher the Glyc. score, the worse was the prognosis of GC. Functional analysis demonstrated that, in samples with a high Glyc. score, several cancer-related signaling pathways, including P53 signaling pathway, cell cycle, and DNA replication pathways, were activated. Activation of the P53 signaling pathway occurs not only through canonical mutations but also through the high-level amplification of the P53 gene (Harris and Levine, 2005). The P53 signaling pathway is associated with resistance to targeted chemotherapies (Joerger and Fersht, 2016) and plays an important role in the occurrence and development of gastroesophageal cancers (Clemons et al., 2013). Cell cycle signaling in tumor cells leads to self-renewal, proliferation, and invasion (Evan and Vousden, 2001). In samples with a higher Glyc. score, the cell cycle signaling pathway was aberrantly activated, which promoted carcinogenesis and the transformation of cancer stem cells. DNA replication pathway plays a role in both tumor suppression and tumor promotion (Li and Heyer, 2008). In this study, we also found that the EMT pathway was activated in the high–Glyc. score group, and DNA replication signaling is reported to promote GC development through the induction of EMT (Wang et al., 2020). Consistently, we found that, in GC, the Glyc. score was significantly high, suggesting that a high Glyc. score might promote GC development through the activation of the abovementioned signaling pathways.

At present, immunological checkpoint blockade of CTLA-4 and PD-L1 has become one of the most promising immunotherapies (Incorvaia et al., 2019). However, some solid tumors respond poorly to immunotherapy (such as pancreatic cancer) (Bear et al., 2020). Studies have shown that the efficacy of tumor immunotherapy is still limited by the highly immunosuppressed TME and the low immunogenicity of cancer cells, which seriously affects the efficacy of treatment (Kishton et al., 2017). In this study, functional enrichment analysis revealed that the high Glyc. score was enriched in metabolic related pathways. Metabolic changes are one of the important characteristics of tumors. To maintain sustained proliferation, tumor cells must adjust their metabolism and nutritional access (Martinez-Outschoorn et al., 2017). Evidence shows that the imbalance of energy metabolism in human body can produce suppressive TME and, thus, inhibit anti-tumor immune response and play a key role in tumor progression and metastasis (Pearce and Pearce, 2013; Andrejeva and Rathmell, 2017; Huby and Gautier, 2021). On the other hand, we found that immune cells in the Glyc. cluster.B, Glyc. gene.cluster.C, and high–Glyc. score group exhibited higher expression levels in patients with GC. Previous studies demonstrated that tumors with immuneexcluded phenotype also showed the presence of abundant immune cells, whereas these immune cells were retained in the stroma surrounding tumor cell nests rather than penetrate their parenchyma. The activation of stroma in TME were considered T-cell suppressive (Chen and Mellman, 2017). Therefore, under this dual effect, the immunotherapy of GC is not ideal.

The ACRG molecular subtype, which focuses on the heterogeneity of GC, comprises four molecular subtypes linked to distinct patterns of molecular alterations, disease progression, and prognosis (Cristescu et al., 2015). In the ACRG analysis, the MSI subtype had the best prognosis, followed by MSS/TP53+ and MSS/TP53−, with the MSS/EMT subtype showing the worst prognosis (Cristescu et al., 2015). Consistent with the survival trends in the ACRG cohort, we found a similar distribution in the Glyc. score; GC samples of the EMT subtype were notably linked to high Glyc. score and had a poorer prognosis. The TCGA molecular subtype, which also focuses on the heterogeneity of GC, comprises four molecular subtypes. In the TCGA analysis, the MSI subtype had the best prognosis, followed by EBV and CIN, with the GS subtype showing the worst prognosis (Cancer Genome Atlas Research Network, 2014). Consistent with the survival trends in the TCGA cohort, we found a similar distribution in the Glyc. score; GC samples of the GS subtype were notably linked to high Glyc. score and had a poorer prognosis.

GC classification methods frequently used in clinical practice, such as Lauren classification and WHO classification, lack a quantitative score for individual patients, and their clinical utility is limited. The molecular subtype systems based on molecular mechanisms and clinical outcomes, such as TCGA subtypes and ACRG subtypes, are easily applicable to the preclinical setting and facilitate clinical decision making. In this study, we attempted to explore the molecular role of glycosylation patterns in GC. We quantified glycosylation modification for each individual patient and developed a similar scoring system, which linked the Glyc. score with the prognosis and immunotherapy of patients with GC.

Our study has some limitations. First, given the individual heterogeneity of GC, the results of our study should be further validated using more multicenter clinical data. Last, our findings have substantial implications for glycosylation of GC, and the detailed molecular mechanisms require further research to explore deeper interactions.

In a conclusion, this study was focused on the analysis of glycosylation in GC, and, on the basis of the modification patterns, we constructed the Glyc. score to explore the extensive regulation mechanisms by which glycosylation modification affect tumorigenesis. The relationship between the scoring system and clinical outcomes in patients with GC was demonstrated, and model validation was performed using several external datasets from the GEO and TCGA databases. This study provides novel insights into the role of glycosylation modification in GC development.

## Data Availability

The datasets presented in this study can be found in online repositories. The names of the repository/repositories and accession number(s) can be found in the article/Supplementary Material.
