# Supplementary figures and images for: Expression Patterns of Glycosylation Regulators Define Tumor Microenvironment and Immunotherapy in Gastric Cancer
Source: Front Cell Dev Biol. 2022 Feb 15;10:811075. doi: 10.3389/fcell.2022.811075 (PMC8886025; doi:10.3389/fcell.2022.811075)

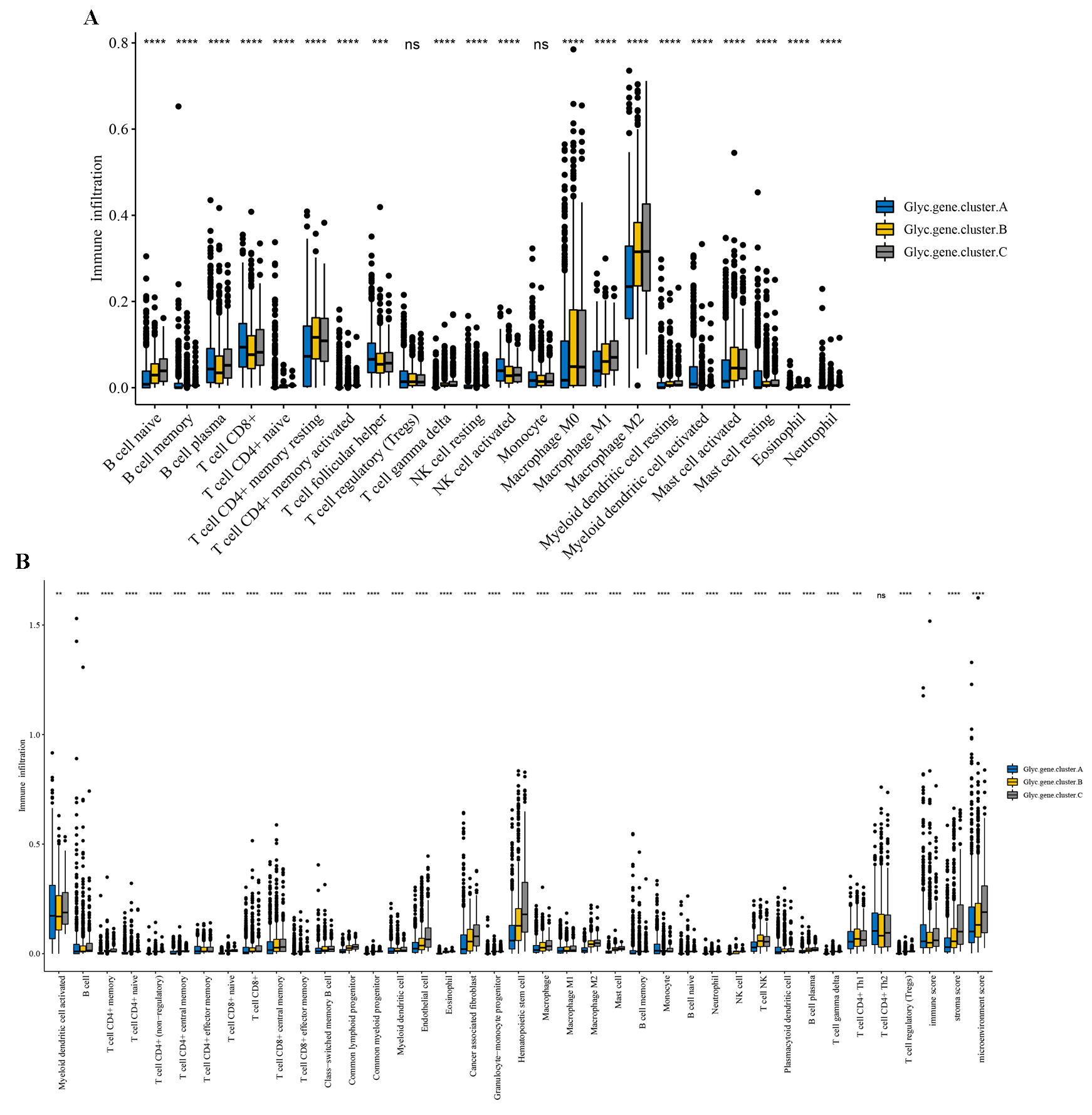

Supplement: Supplementary file 1 [file Image3.JPEG]

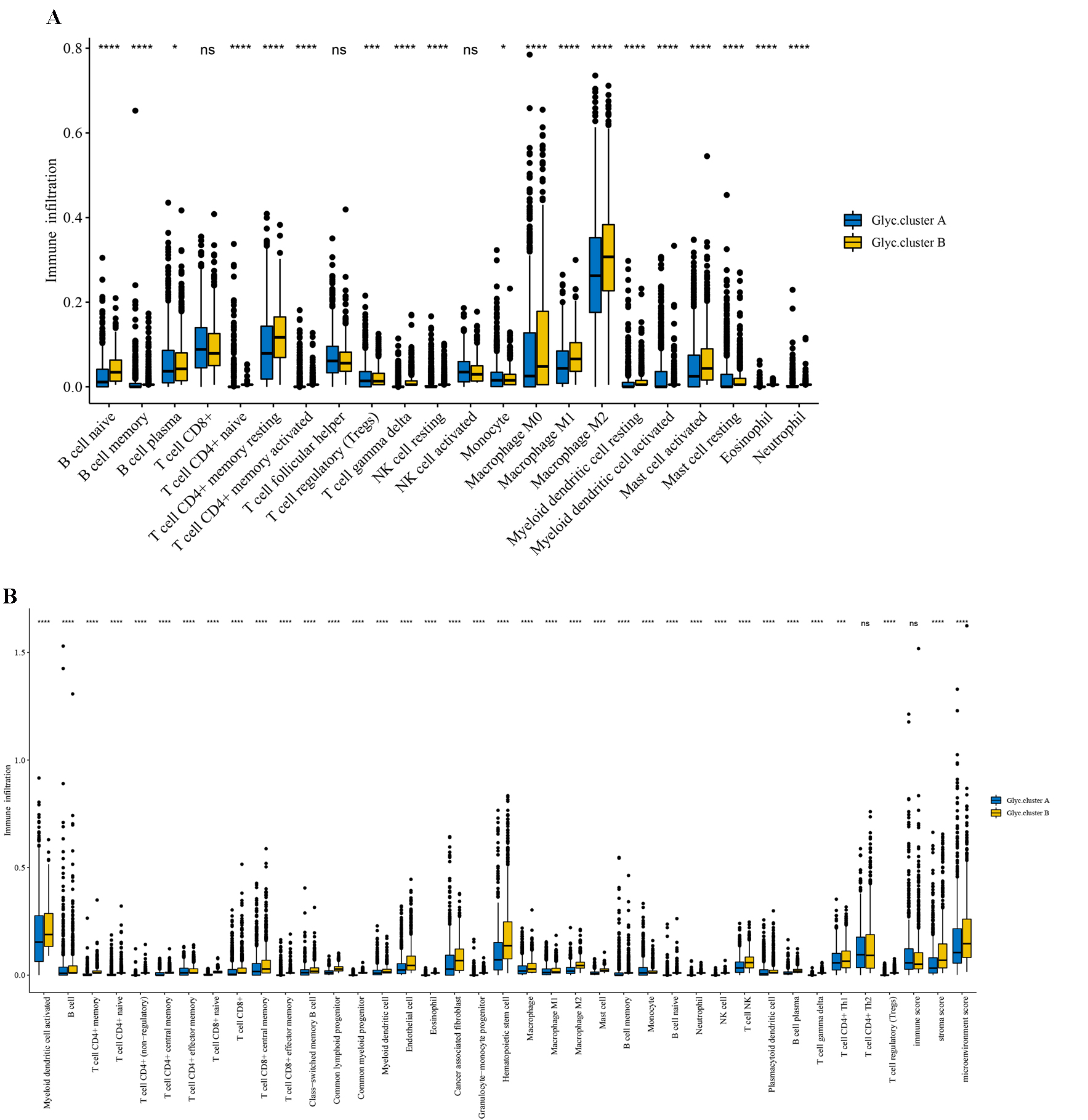

Supplement: Supplementary file 4 [file Image1.JPEG]

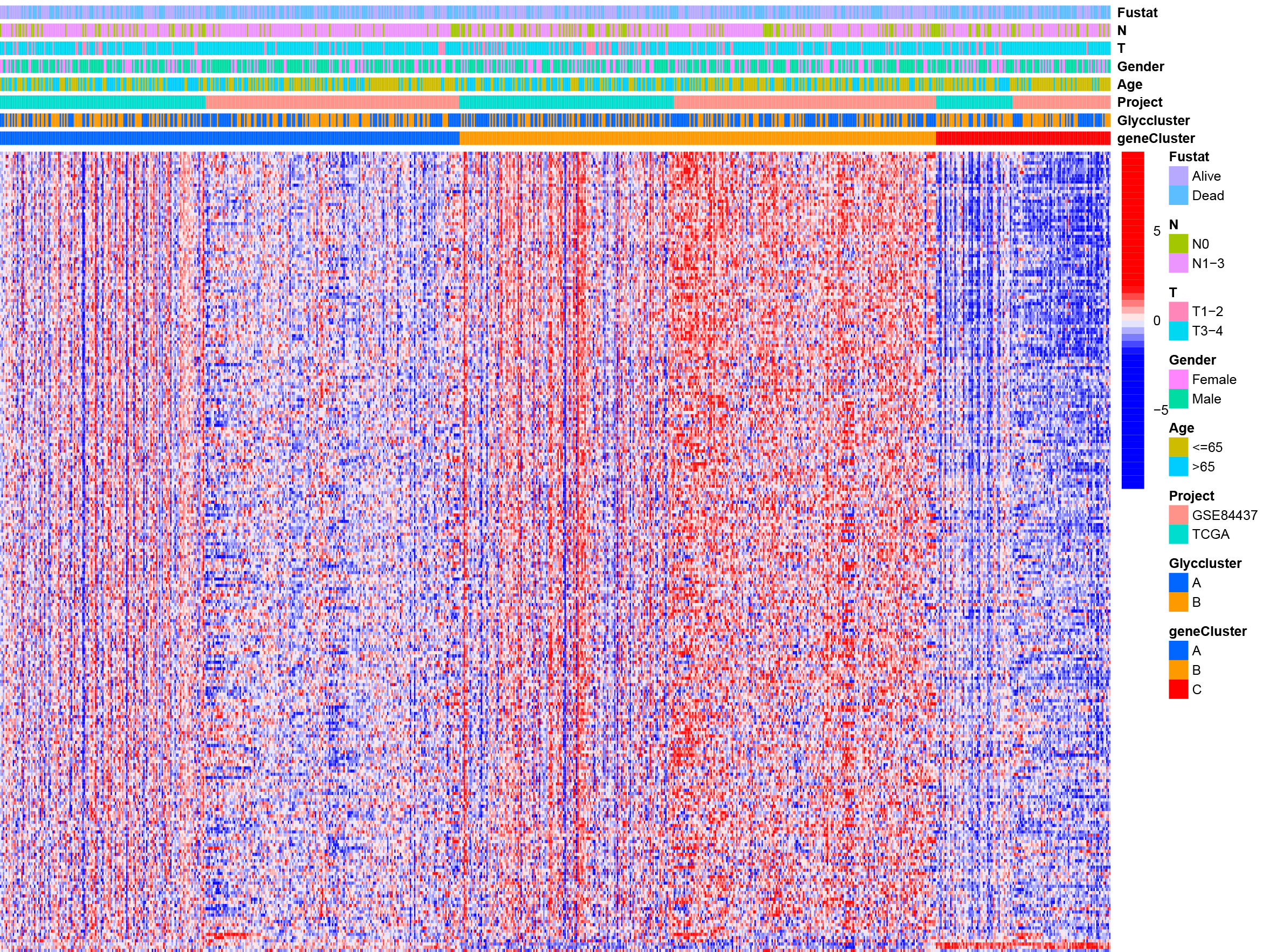

Supplement: Supplementary file 5 [file Image4.JPEG]

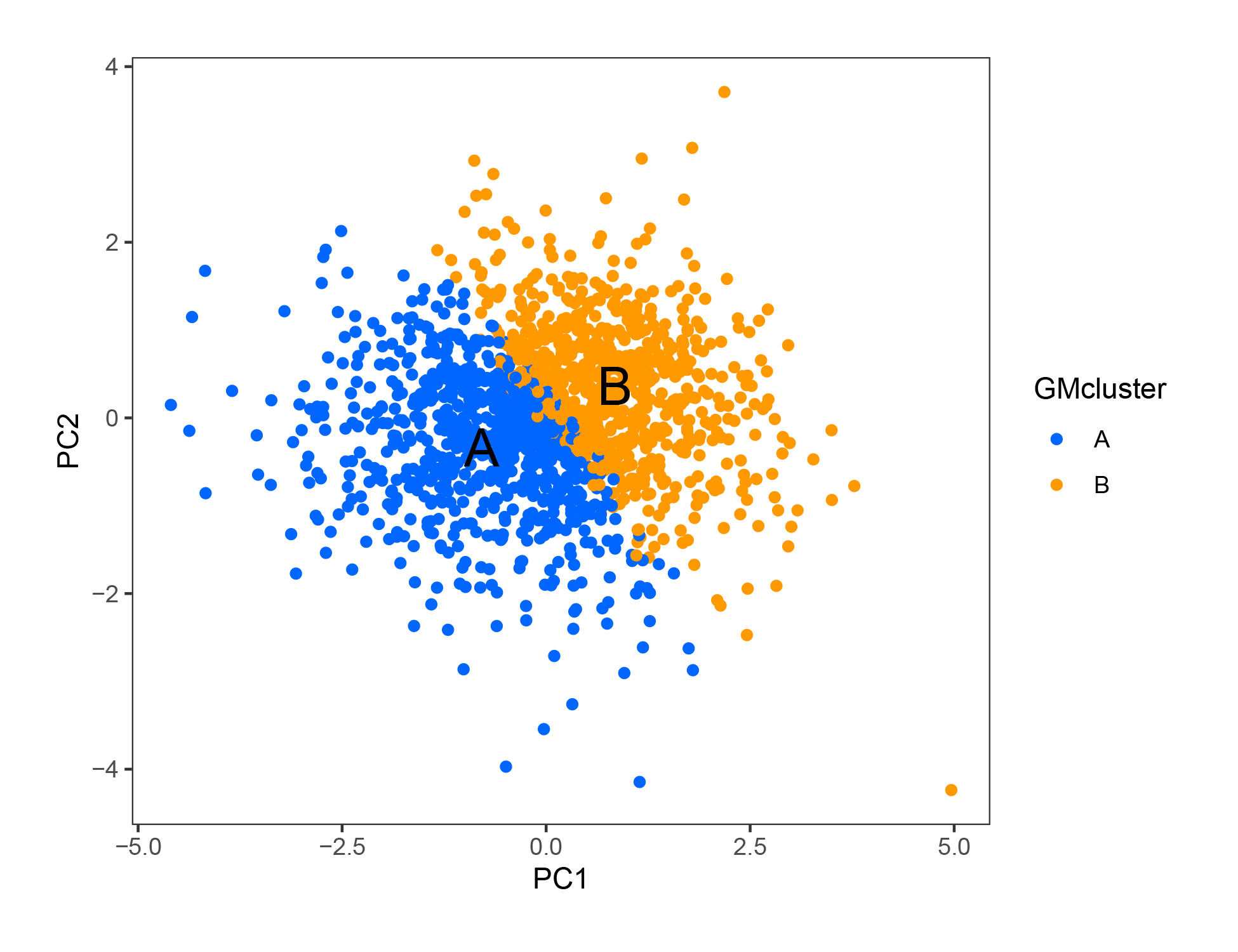

Supplement: Supplementary file 7 [file Image2.JPEG]
